# Ligand-induced protein transition state stabilization switches the binding pathway from conformational selection to induced fit

**DOI:** 10.1073/pnas.2317747121

**Published:** 2024-03-25

**Authors:** Olof Stenström, Carl Diehl, Kristofer Modig, Mikael Akke

**Affiliations:** ^a^Division of Biophysical Chemistry, Center for Molecular Protein Science, Department of Chemistry, Lund University, SE-221 00 Lund, Sweden

**Keywords:** protein dynamics, ligand-binding kinetics, molecular recognition

## Abstract

Protein–ligand complex formation is fundamental to biological function. A central question is whether proteins spontaneously adopt binding-competent conformations to which ligands bind conformational selection (CS) or whether ligands induce the binding-competent conformation induced fit (IF). Here, we resolve the CS and IF binding pathways by characterizing protein conformational dynamics over a wide range of ligand concentrations using NMR relaxation dispersion. We determined the relative flux through the two pathways using a four-state binding model that includes both CS and IF. Experiments conducted without ligand show that galectin-3 exchanges between the ground-state conformation and a high-energy conformation similar to the ligand-bound conformation, demonstrating that CS is a plausible pathway. Near-identical crystal structures of the apo and ligand-bound states suggest that the high-energy conformation in solution corresponds to the apo crystal structure. Stepwise additions of the ligand lactose induce progressive changes in the relaxation dispersions that we fit collectively to the four-state model, yielding all microscopic rate constants and binding affinities. The ligand affinity is higher for the bound-like conformation than for the ground state, as expected for CS. Nonetheless, the IF pathway contributes greater than 70% of the total flux even at low ligand concentrations. The higher flux through the IF pathway is explained by considerably higher rates of exchange between the two protein conformations in the ligand-associated state. Thus, the ligand acts to decrease the activation barrier between protein conformations in a manner reciprocal to enzymatic transition-state stabilization of reactions involving ligand transformation.

## Significance

Proteins change their conformations as part of their biological functions, for example, when binding another molecule (ligand). Two pathways are conceivable: Either the ligand binds preferentially to a rare conformation with higher affinity (“conformational selection”) or the ligand first binds to the dominant conformation and induces a transition to the high-affinity conformation (“induced fit”). We determined all rate constants of these two pathways for lactose binding to the protein galectin-3. Even though galectin-3 exists in a preestablished equilibrium of low- and high-affinity conformations, lactose binds primarily via the induced-fit pathway because lactose lowers the free-energy barrier of galectin-3’s conformational change. As a result, the reaction flux switches from conformational selection to induced fit already at quite low ligand concentrations.

**Fig. 1.**
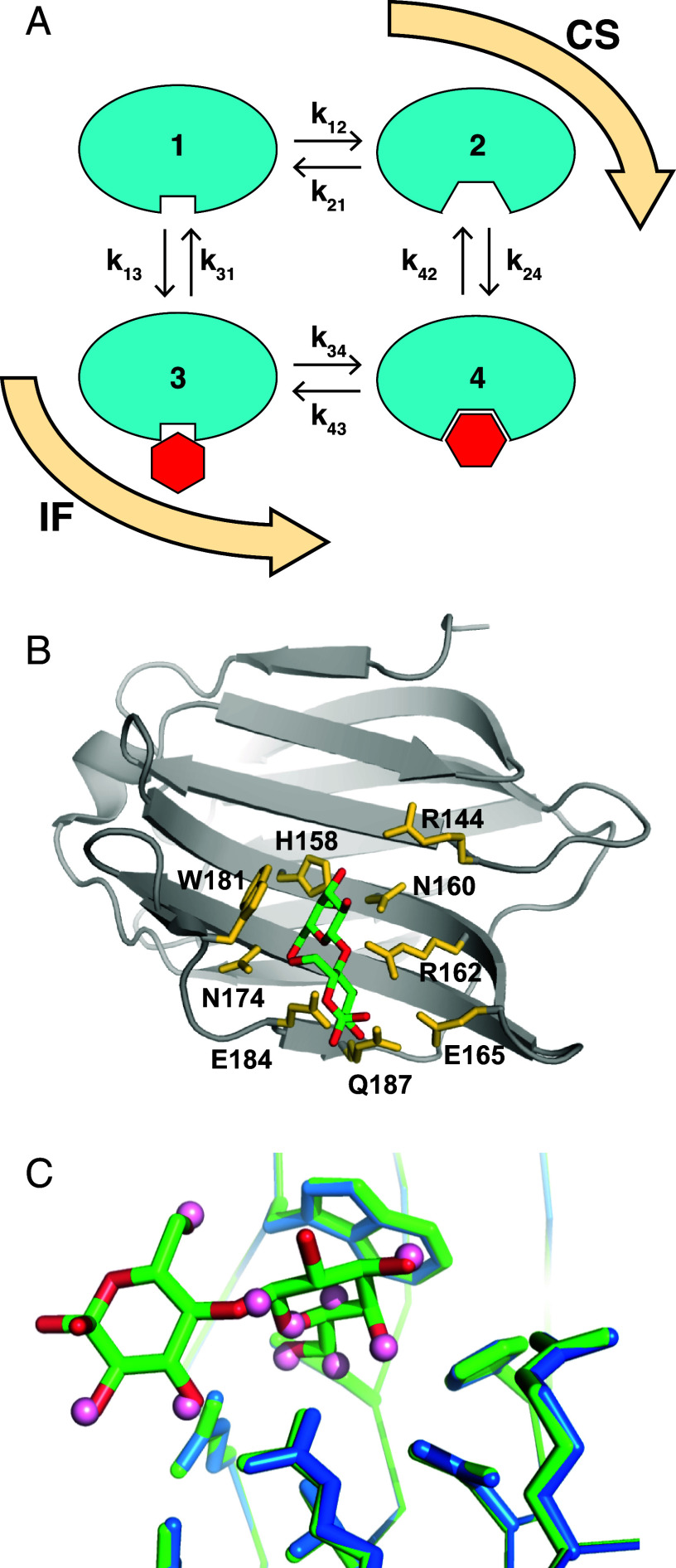
Ligand binding by galectin-3C. (*A*) Schematic outline of the four-state model including the conformational-selection (CS; states 1-2-4) and induced-fit (IF; states 1-3-4) pathways for ligand binding. Free ligand is not shown. State 1 is the binding-incompetent closed state; state 2 is the binding-competent open state; state 3 is the intermediate state in the IF pathway; and state 4 is the final, highest-affinity bound state. *k*_on_ = *k*_13_ = *k*_24_, *k*_off,3_ = *k*_31_, and *k*_off,4_ = *k*_42_. The macroscopic apo state comprises states 1 and 2, while the macroscopic ligand-bound state comprises states 3 and 4. (*B*) Three-dimensional structure of lactose-bound galectin-3C. The backbone trace is shown in gray ribbon representation with ligand-coordinating side chains highlighted in yellow. The lactose molecule is shown in stick representation with carbon atoms in green and oxygen atoms in red. (*C*) Comparison of the binding site in the apo and lactose-bound galectin-3C crystal structures, highlighting the positions of oxygen atoms in water molecules (pink spheres) bound to the apo protein and in lactose (red). Water oxygens primarily match those lactose oxygens that are less exposed to solvent. The protein backbone and side chains are shown in stick representation: blue, apo; green, lactose bound. PDB entities: apo, 3zsl; lactose bound, 3zsj (23). Panels (*B*) and (*C*) were prepared using PyMOL (Schrödinger, LLC).

The mechanism of protein–ligand binding has been central to life science for more than 60 y, ever since the seminal papers on enzyme flexibility and activity (1, 2) and allosteric regulation (3, 4). At the heart of the matter lies the question whether proteins spontaneously adopt conformations complementary to their ligands, to which the ligands subsequently bind, as envisioned by Monod–Wyman–Changeux (4), or whether ligands induce those conformations upon binding, as initially proposed by Koshland (1). These mechanisms of molecular recognition are denoted “conformational selection” (CS; sometimes referred to as “select fit”) and “induced fit” (IF), respectively (Fig. 1*A*). Koshland-Nemethy-Filmer (5) subsequently presented a modified view that allowed for CS as a possible mechanism for ligand binding. IF has dominated the textbook view of protein–ligand interactions for a long time. However, research over the past decades increasingly supports CS as an alternative to the IF paradigm (6, 7). In particular, recent developments in NMR spectroscopy and computational chemistry have provided evidence of short-lived, weakly populated conformers of the nonbound protein that resemble the ligand-bound state (8–18). While these results suggest that ligand binding might proceed via the CS pathway, they do not in themselves provide any direct evidence that this is the case. It is the net reaction flux, rather than the rates and equilibrium populations of individual reaction steps, that determines which pathway is the dominating one (18–22).

NMR relaxation dispersion experiments and line-shape analysis can probe conformational transitions that give rise to changes in the chemical shift of individual nuclei, which provides a powerful means of monitoring ligand-binding kinetics under equilibrium conditions (8, 24–33). The results yield both an atomic-resolution view of the binding process, as sensed by the protein, and the microscopic rate constants of the individual steps of the reaction. Recent work has presented analytical expressions for chemical exchange processes involving an arbitrary number of states (34, 35). Previous studies have derived detailed expressions for each of the IF and CS pathways that make it possible to distinguish between these pathways based on the kinetic rate constants (36–42). However, it is important to recognize that the two pathways are not mutually exclusive but can both contribute simultaneously to the overall reaction flux (41, 43).

Here, we use NMR relaxation dispersion experiments to measure the underlying protein dynamics and determine the relative importance the CS and IF pathways for lactose binding to the carbohydrate-binding domain of human galectin-3 (galectin-3C). Galectin-3C binds saccharides and synthetic ligands in a shallow, solvent-exposed groove on one side of its β-sandwich structure (23); see Fig. 1*B*. We performed ^15^N CPMG relaxation dispersion measurements at variable degrees of ligand saturation and analyzed the data using a four-state model that includes both the CS and IF pathways (Fig. 1*A*). We found that ligand-free (apo) galectin-3C transiently adopts an “open” conformation similar to the ligand-bound state, indicating that the protein is capable of binding ligand via the CS pathway. Indeed, the determined rate constants reveal that the ligand has a higher affinity for the open conformation than for the closed one. Yet, the IF pathway dominates across the entire range of ligand concentrations used here, corresponding to saturation levels between 4 and 76%. This result is explained by significantly higher exchange rates between states 3 and 4 than between 1 and 2, see Fig. 1*A*, which implies that the ligand stabilizes the transition state of the protein conformational change.

## Results and Discussion

We investigated the kinetic ligand-binding mechanisms in galectin-3C by recording ^15^N CPMG relaxation dispersion experiments on the apo protein and seven additional samples with increasing amounts of added ligand; the resulting relaxation rate constants have been deposited in the Biological Magnetic Resonance Data Bank (44) under accession numbers 52174 and 52175. By numerically fitting the CPMG data globally to a four-state model, using the Bloch–McConnell equations (45), we included both the CS and IF pathways of ligand binding. We analyzed the data in two steps: First, we fitted the data for the apo state separately using a two-state model and then proceeded to fit the concentration dependent data, including the results from the apo fit as fixed parameters. Fitting the four-state model is tractable in the present case, due in part to the distinctive variation in dispersion profiles with ligand concentration, and because one of the four equilibria can be isolated and fully determined from a two-state fit of the apo state dynamics, which reduces the roughness of the target function (46).

### Apo Galectin-3C Transiently Adopts a Conformation Similar to the Ligand-Bound Conformation.

**Fig. 2.**
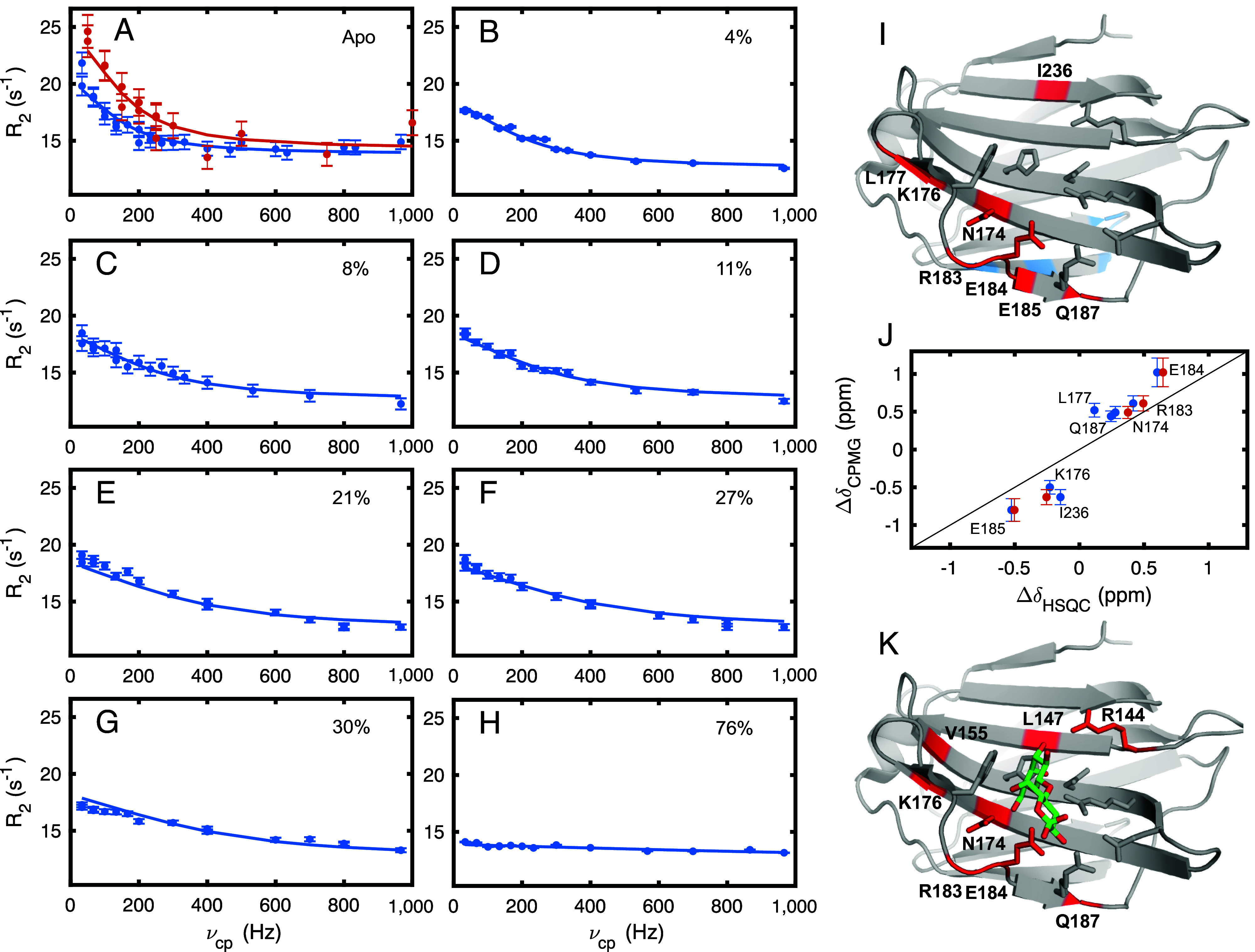
Conformational exchange and ligand-binding dynamics in galectin-3C. (*A*–*H*) Representative ^15^N CPMG relaxation dispersion data for residue E184. *SI Appendix*, Fig. S1 shows the corresponding data for all residues included in the fits. Error bars correspond to ±1 SD. (*A*) Relaxation dispersions for apo galectin-3C, with blue (red) symbols indicating data obtained at a static magnetic field strength of 14.1 T (18.8 T). (*B*–*H*) Relaxation dispersions obtained at 14.1 T for galectin-3C with varying levels of lactose saturation: (*B*) 4%, (*C*) 8 %, (*D*) 11 %, (*E*) 21 %, (*F*) 27 %, (*G*) 30 %, and (*H*) 75 %. (*I*) Residues showing conformational exchange in apo galectin-3C mapped onto the structure. Red indicates residues in the binding site showing slower exchange dynamics between states 1 (major population) and 2 (minor population). Blue indicates residues showing fast exchange dynamics unrelated to the exchange between states 1 and 2 (all located on the backside of the protein, away from the binding site). Side chains involved in ligand coordination are shown in stick representation. (*J*) Chemical shift differences between states 1 and 2, Δδ_CPMG_, in apo galectin-3C determined from ^15^N CPMG relaxation dispersion experiments plotted versus chemical shift differences between the apo and lactose-bound forms, Δδ_HSQC_, determined either directly from HSQC spectra of each form (blue symbols; Pearson correlation coefficient: *r* = 0.96; slope = 1.82) or from the chemical shifts obtained by fitting the four-state model (red symbols; *r* = 0.99; slope = 1.47). A straight line with slope = 1 is drawn to guide the eye. (*K*) Residues colored red show conformational exchange in all seven samples with lactose added. Panels (*I*) and (*K*) were prepared using PyMOL (Schrödinger, LLC) and PDB entry 3zsj (23).

We first investigated the conformational dynamics of galectin-3C in the absence of ligand. We recorded ^15^N CPMG relaxation dispersion experiments on apo galectin-3C at two static magnetic field strengths, 14.1 T and 18.8 T. Significant exchange contributions to the transverse relaxation rates were observed for 13 residues in apo galectin-3C (Fig. 2*A* and *SI Appendix*, Fig. S1*A* and Table S1; BMRB entry 52174 (47)). Residue-specific fits reveal exchange processes on two distinct timescales. One set of six residues (V189, V213, D215, A216, L219, and Y221) shows fast exchange dynamics between two states, *k*_ex_ = (4.8 ± 0.8) × 10^3^ s^−1^ and *p*_Major_ = 0.99 ± 0.01. These residues are all located in β-strands 8 to 9 and the connecting hairpin that form the edge of the β-sheet on the opposite side of the protein from the ligand-binding site (Fig. 2*I*). Another set of eight residues, viz. N174, K176, L177, R183, E184, E185, Q187, and I236 shows slower exchange rates, *k*_12_ = 34 ± 4 s^−1^ and *k*_21_ = 735 ± 80 s^−1^, and relative populations, *p*_1_ = 0.96 ± 0.02 and *p*_2_ = 0.04 ± 0.02. Except for I236, these residues are all located in β-strands 5 to 6 that form part of the binding site; I236 is located >5 Å from the binding site in β-strand 11.

The chemical shift difference, Δδ, between exchanging states can be extracted from the CPMG dispersions (48, 49), see *SI Appendix*, Table S2. Notably, for the eight residues in the binding site, the chemical shifts of the high-energy conformation of apo galectin-3C are similar to those of lactose-bound galectin-3C, as evidenced by comparing Δδ_CPMG_ obtained from the CPMG relaxation dispersion data and Δδ_HSQC_ measured from HSQC spectra of the apo and lactose-bound states (Fig. 2*J*); Pearson’s correlation coefficient *r* = 0.96. In contrast, none of the exchanging residues located on the backside of the protein experience any significant chemical shift perturbation upon lactose binding; |Δδ_HSQC_| ≤ 0.025 ppm, further indicating that these residues are involved in a separate exchange process unrelated to ligand binding. Note that we do not expect a perfect correlation in Fig. 2*J* because minor conformational adjustments upon binding and the presence of the lactose molecule in the binding site are expected to give rise to additional chemical shift changes. The good agreement between Δδ_CPMG_ and Δδ_HSQC_ provides strong evidence that apo galectin-3C transiently samples conformations resembling the lactose-bound state. As shown previously, the crystal structures of apo and lactose-bound galectin-3C are virtually identical (23). Assuming that lactose-bound galectin-3C adopts the same conformation in solution as in the crystal, the highly similar chemical shifts of the apo and lactose-bound states suggest that the high-energy conformation of apo galectin-3C in solution corresponds to the apo crystal structure. In the apo crystal structure, several water molecules are present in the ligand binding site with their oxygen atoms occupying the same positions as oxygens in the bound lactose. Furthermore, the ligand-coordinating protein side chains have the same orientation in the apo and lactose-bound crystal structures (Fig. 1*C*). These observations explain the similarity in chemical shifts (Fig. 2*J*).

We conducted additional experiments to validate this result and refute the possibility that there might be residual lactose present in the apo sample that would result in exchange between a major population of the apo state and a minor population of the lactose-bound state (see *SI Appendix* for details). Taken together, our results demonstrate that apo galectin-3C exchanges between a “closed” (binding-incompetent) ground state and an “open” (binding-competent) high-energy state, implying that ligand binding can occur via conformational selection. Similar results have been reported for several other proteins (8–11). The exchange observed for apo galectin-3C defines the equilibrium between state 1 (ground state) and state 2 (high-energy state) of the four-state model, *K*_2,1_ = *k*_12_/*k*_21_ = *p*_2_/*p*_1_. These parameters were subsequently fixed when fitting the full four-state model as described below.

### Mapping Binding Pathways by Relaxation Dispersion Measurements at Variable Ligand Concentration.

To resolve the CS and IF binding pathways and determine the flux through each pathway, we measured ^15^N CPMG relaxation dispersion on galectin-3C at seven different concentrations of lactose, covering saturation levels between 4% and 76%. Five of the eight residues that exhibit exchange in the absence of lactose also display relaxation dispersions of sufficient quality at all seven concentrations of lactose: N174, K176, R183, E184, and Q187. Three additional residues, R144, L147, and V155, show exchange for all lactose concentrations and were also included in the analysis (Fig. 2*K*). The relaxation dispersion data vary significantly with the concentration of lactose (Fig. 2 *B*–*H* and *SI Appendix*, Fig. S2; BMRB entry 52175 (50)), as a consequence of the changing populations and the progressively faster on-rate.

Fig. 1 defines the four-state model with the CS and IF pathways connecting states 1-2-4 and 1-3-4, respectively. In fitting the four-state model, we include three global parameters in addition to residue-specific chemical shifts and *R*_2,0_ values. The three global parameters are the on-rate constant for ligand binding, *k*_on_ = *k*_13_ = *k*_24_, the ratio of the rate constants for ligand release from states 3 and 4, *ρ*_off_ = *k*_off,3_/*k*_off,4_, and the ratio of the rate constants for protein conformational change from open to closed in each pathway, *ρ*_close_ = *k*_21_/*k*_43_. All rate constants (and relative populations) can be calculated from these parameters and the dissociation constant, *K*_d_, measured by ITC, together with *k*_12_ and *k*_21_, determined from CPMG dispersion experiments on the apo state (see *Materials and Methods* and *SI Appendix* for details). The assumption that the on-rate constants (*k*_13_ and *k*_24_) of the two pathways are equal is expected to hold in the present case for the following reasons. First, the NMR experiments detect ligand-induced chemical shift changes of residues located in the ligand-binding site. Thus, the on-rate constant measured here includes processes such as the diffusion of the ligand across the protein surface following encounters where the ligand contacts the protein at locations remote from the binding site. Second, the open and closed conformations of galectin-3C are very similar, as evidenced by the fact that chemical shift changes between the apo and lactose-bound states (Δδ_HSQC_) are limited and observed only for residues in the binding site. In the absence of large-scale conformational changes, it is unlikely that the on-rate constant will differ between the two conformations, especially since lactose binding does not involve interactions between charged species. The assumption of equal on-rate constants is not expected to be generally valid for protein–ligand systems.

To fit the four-state model to the CPMG dispersion data, we initially performed grid searches on *k*_on_, *ρ*_off_, and *ρ*_close_, while fitting the chemical shifts and *R*_20_ values, so as to characterize the χ^2^ space. Projections of the χ^2^ space onto two dimensions (i.e., pairs of *k*_on_, *ρ*_off_, and *ρ*_close_; see *SI Appendix*, Fig. S3) clearly show that χ^2^ has a well-defined minimum for 0.3 < *ρ*_close_ < 0.4, whereas the model is less sensitive to *ρ*_off_, although there is a preference for 200 < *ρ*_off_ < 600. Furthermore, we find that low χ^2^ values are obtained with 150 × 10^6^ s^−1^M^−1^ < *k*_on_ < 250 × 10^6^ s^−1^M^−1^. We then performed free fits of all parameters, starting from values of *k*_on_, *ρ*_off_, and *ρ*_close_ corresponding to low χ^2^. The model parameters were fitted simultaneously to all relaxation data, comprising in total 8 × 7 dispersion curves, each containing 15 to 24 data points. The best-fit results yielded *k*_on_ = (172 ± 37) × 10^6^ s^−1^M^−1^, *ρ*_close_= 0.43 ± 0.01, and *ρ*_off_ = 266 ± 48. From these fitted parameter values, we then extracted the off-rate constants, *k*_off,3_ = (5.5 ± 1.9) × 10^5^ s^−1^ and *k*_off,4_ = (2.1 ± 0.5) × 10^3^ s^−1^, and the rates of conformational exchange involving the macroscopic bound state, *k*_43_ = (1.93 ± 0.05) × 10^3^ s^−1^ and *k*_34_ = (27 ± 5) × 10^3^ s^−1^. Since *k*_12_ and *k*_21_ are known from the experiments conducted on apo galectin-3C, described above, the four-state model is thus completely described (Table 1).

**Table 1.** Rate constants describing the four-state model

Rate constant^*^	Fitted value	Estimated error
*k*_12_ (s^−1^)	34	4
*k*_21_ (s^−1^)	735	80
*k*_on_ (10^6^ M^−1^ s^−1^)	172	37
*k*_off,3_ (10^5^ s^−1^)	5.5	1.9
*k*_off,4_ (10^3^ s^−1^)	2.1	0.5
*k*_34_ (10^3^ s^−1^)	27	5
*k*_43_ (10^3^ s^−1^)	1.93	0.05

^*^k_off,3_, *k*_off,4_, *k*_34_, and *k*_43_ were calculated from the fitted parameters *ρ*_off_ and *ρ*_close_; see *Materials and Methods* and *SI Appendix* for details.

For comparison with the fitted value of *k*_on_, we estimated the diffusion-limited on-rate constant for lactose binding using Eq. **5**, which yielded *k*_onD_ = 10 × 10^9^ s^−1^M^−1^. Estimates that take the relative size of the binding site into account (51, 52) are reduced by roughly a factor of 10 from this value. Thus, the experimental value of *k*_on_ is significantly lower than *k*_onD_, which is expected because the former includes not only the diffusion-limited rate of encounter but also the diffusive “search” by the ligand across the protein surface from the initial point of contact to the immediate neighborhood of the binding site. This result is also in keeping with our recent work, showing that several hundred ligand–galectin-3C encounters occur for each successful binding event (53).

### Ligand Affinity Is Higher for the Open State.

The fitted rate constants enable us to determine the affinity of each protein conformation (open or closed) for lactose, which is not available from traditional binding assays, including isothermal titration calorimetry or fluorescence (54, 55). The rate constants establish that the lactose affinity is considerably higher for state 2 than for state 1, *K*_a,2_/*K*_a,1_ = *k*_off,3_/*k*_off,4_ = 270, in keeping with the notion that state 2 has an open, binding-competent conformation, whereas state 1 is closed and binding-incompetent, as required in the CS binding model. These results might give the (incorrect) impression that the CS pathway dominates—however, at this level of interpretation, we have not yet considered the critical determinant, namely the flux through each pathway (19, 20).

### The Induced-Fit Pathway Dominates Despite the Presence of a Bound-Like Conformation in the Apo State.

**Fig. 3.**
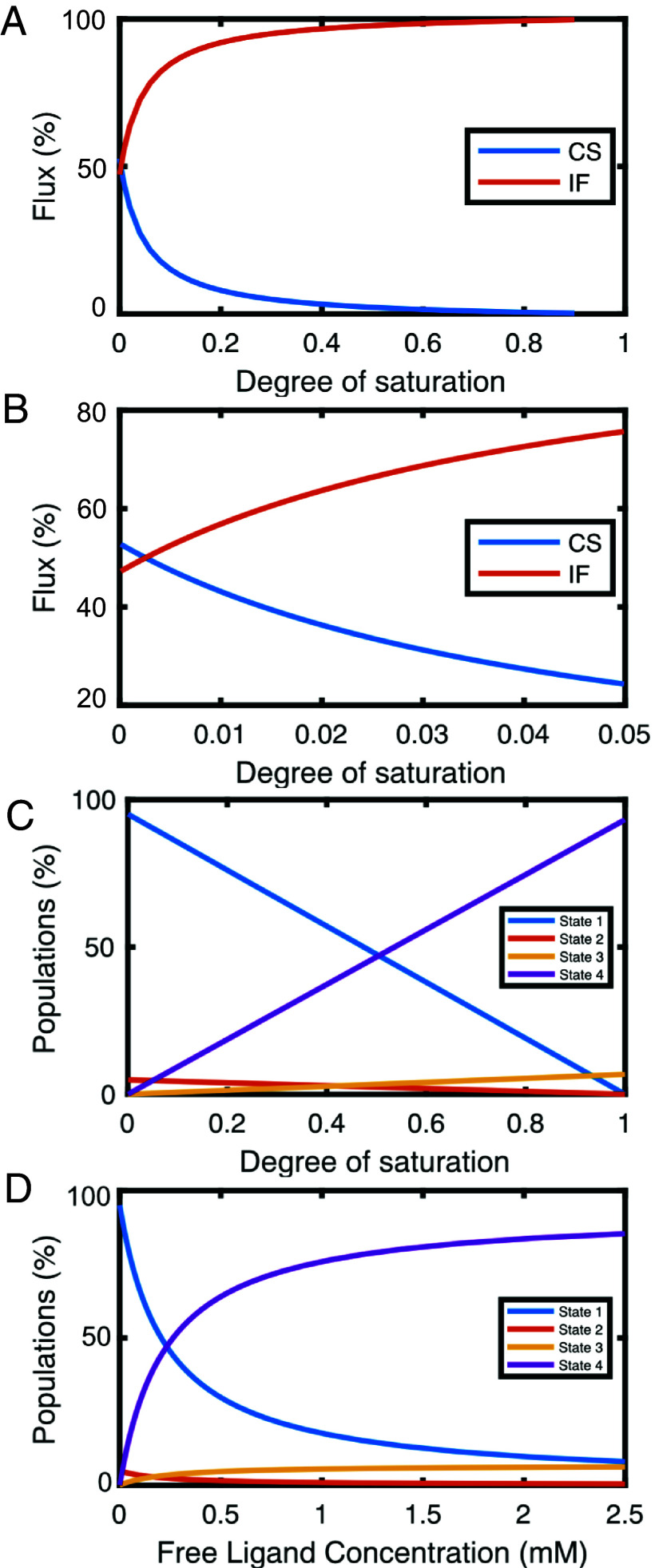
Fluxes and populations as a function of the degree of saturation and ligand concentration. (*A* and *B*) Fluxes through the CS (blue) and IF (red) pathways, plotted versus the degree of saturation. (*B*) Close-up view of the flux at low degrees of saturation, showing the cross-over between the CS and IF pathways. (*C* and *D*) Relative populations of states 1 to 4, plotted versus the degree of saturation (*C*), or the free ligand concentration (*D*). Blue, state 1; red, state 2; yellow, state 3; and magenta, state 4.

Next, we calculated the fluxes through the CS and IF pathways as a function of lactose concentration based on the determined rate constants; see Eqs. **6** and **7**, which show that the flux through a given pathway is the inverse of the sum of the “waiting times” at each intermediate state. The results reveal that the IF pathway dominates under all conditions used in the present experiments (Fig. 3*A*). The flux through the IF pathway varies between 73% of the total flux at the lowest ligand concentration, [*L*]_tot_ = 29 µM (4% saturation), to 99% at the highest ligand concentration, [*L*]_tot_ = 0.95 mM (76% saturation). By extrapolation, we find that the binding pathway switches from CS to IF when the ligand concentration becomes greater than 2 µM, where the relative population of bound states is only 0.3% (Fig. 3*B*). This result reinforces the notion that the existence of exchange between closed (binding-incompetent) and open (binding-competent) conformations of the apo protein does not in and of itself imply that CS is the dominant binding pathway (19, 20), even though the open conformation has a higher affinity for ligand, as shown here. Rather, the conformational exchange in the free state simply reflects the fact that the two conformations differ in free energy by a few *k*_B_*T* and are separated by relatively moderate free energy barriers that can be readily crossed at ambient temperature, as expected for a protein that changes its conformation as part of its biological function.

**Fig. 4.**
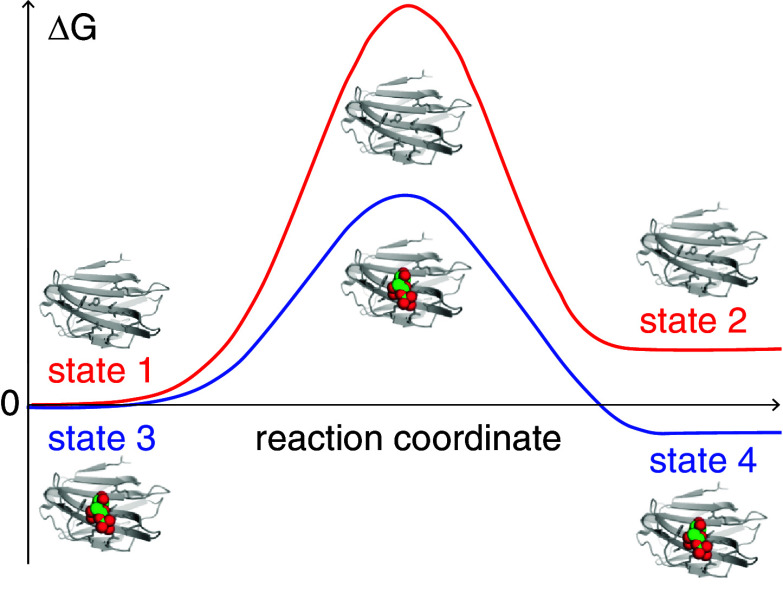
Ligand-induced transition-state stabilization of the closed–open conformational transition. Schematic, qualitative view of the free energy profile along the reaction coordinate of the closed–open conformational transitions. The curves illustrate the transition between states 1 and 2 (red, without ligand) and between states 3 and 4 (blue, with ligand present). The free energies of the closed states (1 and 3) have been set to the same value (Δ*G* = 0) for ease of comparison. The figure is not intended to give a quantitative representation of the relative free energies of the different states.

The dominance of the IF binding pathway is explained by a significantly higher rate of transition from closed to open conformation in the ligand-bound form, *k*_34_ = 27 × 10^3^ s^−1^, than in the apo form, *k*_12_ = 34 s^−1^, cf. Fig. 1. Importantly, this result indicates that the ligand decreases the barrier between the two protein conformations (Fig. 4), which provides direct validation of the mechanism for ligand-induced conformational change hypothesized by Koshland–Nemethy–Filmer (5); similar results have previously been reported for the case of coupled folding and binding (41). We recognize that this phenomenon is reciprocal to enzymatic transition-state stabilization in reactions that change the ligand conformation or chemical structure. Thus, the effect is fully expected from a physicochemical perspective, although it is arguably a much less commonly acknowledged concept than enzymatic transition-state stabilization.

Fig. 3 *C* and *D* show how the relative populations of states 1 to 4 vary with lactose concentration and degree of saturation. State 2 dominates over state 3 in the regime below a protein saturation level of 42% (Fig. 3*C*), but again at least 97% of the total flux goes through the IF pathway under these conditions. The macroscopic lactose-bound form is dominated by state 4, but a non-negligible fraction of ligand-bound protein is in state 3, *p*_3_:*p*_4_ = 0.07:0.93. It is conceivable that the relatively weak affinity of lactose leads to the detectable population of closed conformations in the macroscopic lactose-bound form, whereas it might be expected that high-affinity ligands drive the system further toward state 4 (56). Given that *p*_3_:*p*_4_ is non-negligible, we reevaluated the chemical shift comparison in Fig. 2*J* by plotting Δδ_CPMG_ (=|δ_1_ – δ_2_|) against Δδ_14_ = |δ_1_ – δ_4_|, instead of Δδ_HSQC_ = |δ_apo_ – (*p*_3_δ_3_ + *p*_4_δ_4_)/(*p*_3_ + *p*_4_)|. The corrected chemical shift differences are represented by the red symbols in Fig. 2*J*, which show improved agreement between the two datasets (*r* = 0.99), further bolstering the conclusion that apo galectin-3C samples bound-like conformations.

### The Four-State Model Resolves Conformation-Specific Chemical Shifts.

The chemical shifts determined for each residue included in the four-state model are listed in *SI Appendix*, Table S3. For all residues but one, the chemical shifts of states 2 (δ_2_) and 4 (δ_4_) are more similar to one another than they are to either of δ_1_ or δ_3_. These results are perfectly in line with the conformational changes expected for the CS and IF pathways (cf. Fig. 1) but are not presumed when constructing the four-state model. We note that for all residues but E184, the conformational change from state 3 to 4 results in a greater change in chemical shift than does binding of lactose to the open conformation (i.e., the change from state 2 to 4). This result might also be expected since the chemical shift is arguably more sensitive to local and distinct conformational changes than to low-affinity binding of lactose onto the closed conformation. Thus, the overall picture emerging from the chemical shifts extracted by fitting the four-state model makes intuitive sense.

### Relaxation Dispersion Profiles Are Diagnostic of the Binding Mechanism.

**Fig. 5.**
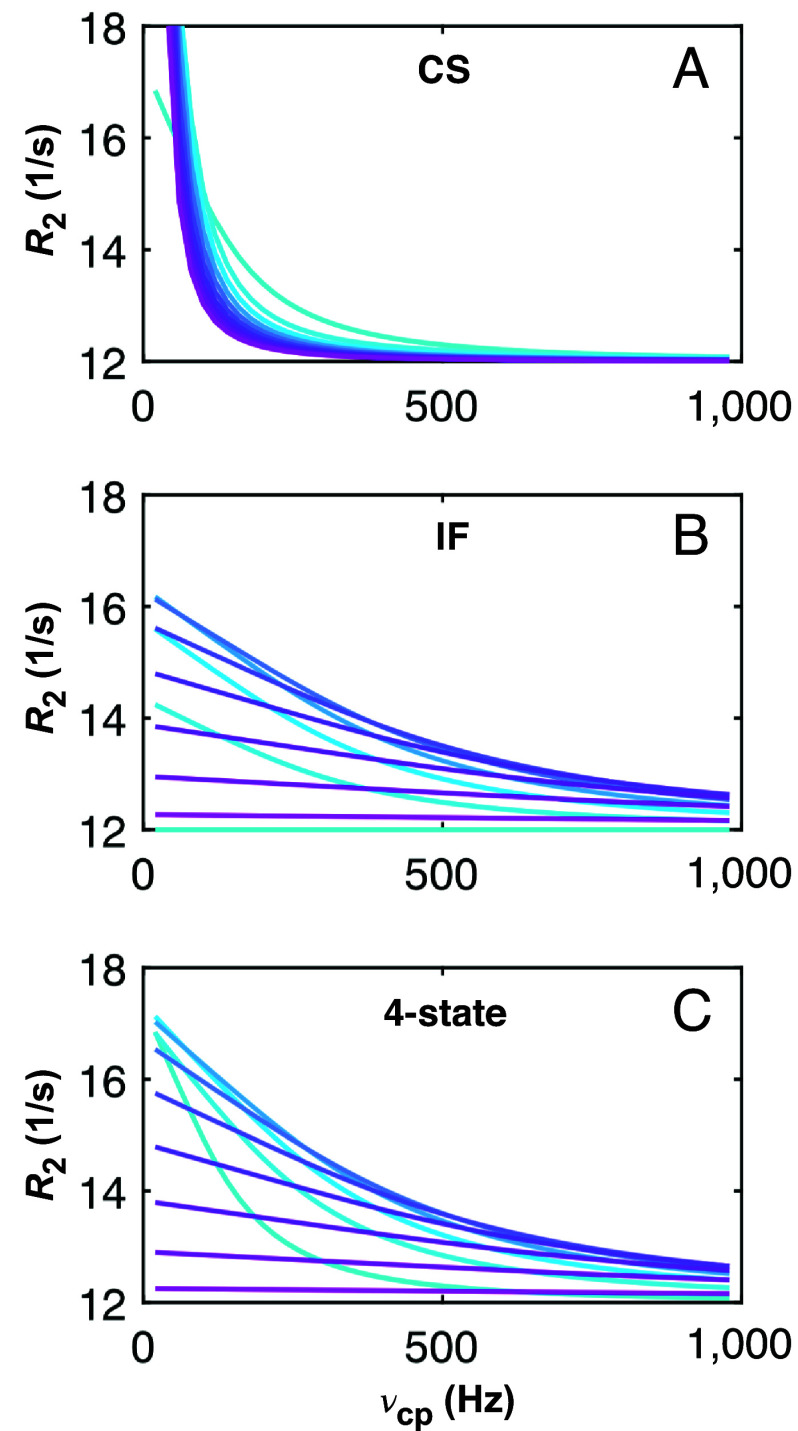
Relaxation dispersion profiles as a function of ligand concentration for different binding mechanisms. (*A*) CS binding pathway. (*B*) IF binding pathway. (*C*) The four-state binding model comprising both CS and IF. The individual curves show relaxation dispersions for protein samples with different extents of ligand saturation, indicated by the color scheme going from light blue for 0% saturation to magenta for 90% saturation, in steps of 10%; in total 10 curves. In panel (*A*), with increasing ligand saturation, the dispersion curves reach higher *R*_2_ values at ν_cp_ = 0 and show a sharper decline at higher ν_cp_. In panel (*B*), the dispersion profile is flat at 0% saturation, then first increases with increasing saturation, followed by a decrease to again reach a flat plateau. In panel (*C*), the dispersion profiles resemble those in panel (*B*) but start out with a significant dispersion at 0% saturation [identical to the case in panel (*A*)]. The figure was generated by simulating relaxation dispersions for the different binding mechanisms using the rate constants determined herein for lactose binding to galectin-3C.

Fig. 5 compares relaxation dispersion curves for the full four-state model with those expected for purely CS or IF binding mechanisms, using for each pathway the relevant exchange parameters resulting from the fit of the four-state model to our experimental data. Clearly, the dispersion curves describing the four-state model, which is a representation of the underlying experimental data, are very different from the pure CS or IF scenarios. The simulated CPMG relaxation dispersion profiles reveal diagnostic signatures for distinguishing between the different reaction mechanisms, as has recently been demonstrated for the IF and CS pathways by the Weikl and Griesinger groups (42). For example, when the CS pathway dominates, *R*_2_ decreases progressively with increasing lactose concentration, but the dispersion profiles maintain a noticeable upward bend to higher *R*_2_ values at low *ν*_cp_, even for the highest [*L*] (Fig. 5*A*). By contrast, when the IF pathway dominates, *R*_2_ starts out at low values and first increases progressively with increasing [*L*], while flattening out at high [*L*] (Fig. 5*B*). The experimental data are effectively a mixture of the dispersion profiles expected for pure IF and CS binding pathways, in that the profiles progressively decrease in *R*_2_ and flatten out with increasing [*L*], without showing any upward bend at low *ν*_cp_ (Fig. 5*C*). Thus, the dispersion profiles describing lactose binding to galectin-3C indicate that exchange between all 4 states must be taken into account, even though the flux through the CS pathway is negligible at higher [*L*]. This result is explained by the fact that the exchange between states 1 and 2 (a key feature of the CS model) exist in conjunction with the IF pathway. That is, in the case of lactose binding to galectin-3C, the four-state exchange predominantly follows a linear exchange mechanism: 2 ⇌ 1 ⇌ 3 ⇌ 4, where state 1 is dominant at low ligand concentrations, whereas the step 2 ⇌ 4 does not contribute appreciably to the overall flux (cf. Fig. 1*A*).

### Concluding Remarks.

We have shown that protein NMR relaxation dispersion experiments can resolve the CS and IF ligand-binding pathways, even in those cases where both contribute to binding. By fitting a four-state binding model comprising both the CS and IF pathways, we determined the individual rate constants that provide information on conformation-specific ligand affinities, as well as the relative flux through the two pathways. Our results reveal that galectin-3C binds lactose almost exclusively via the IF pathway, even though the apo protein populates bound-like conformations with higher ligand affinity. The dominance of the IF pathway arises primarily from a faster rate of protein conformational change in the lactose-associated form than in the apo form. Thus, the ligand acts to lower the transition barrier for protein conformational dynamics, a phenomenon reciprocal to enzymatic transition-state stabilization. Our results provide an enriched view of induced fit and conformational selection that extends current understanding of protein–ligand binding processes.

## Materials and Methods

### Protein Expression and Sample Preparation.

^15^N-labeled galectin-3C samples were expressed, purified, and prepared as described (54, 57). Following an initial set of CPMG experiments on the original apo galectin-3C sample, the sample was filtered using a Vivaspin 2 MWCO 5,000 centrifugal concentrator to dilute by a factor of 10^4^ any lactose possibly remaining in the original sample. Lactose titrations were carried out by titrating small aliquots of lactose (8.8 mM or 35 mM lactose dissolved in NMR buffer) into three different galectin-3C samples, resulting in seven samples covering 7 to 75% saturation of the protein with ligand (see *SI Appendix*, Table S4 for detailed information on sample concentrations). ^15^N CPMG relaxation dispersion datasets were acquired at each titration point.

### Relaxation Dispersion Experiments.

Relaxation-compensated CPMG ^15^N relaxation dispersion experiments (58) were performed at static magnetic field strengths of 14.1 T (apo and lactose-containing samples) and 18.8 T (apo sample only) and a temperature of 301 ± 0.1 K, which was calibrated using a methanol sample before each series of experiments. An equilibration period of 5 ms was added prior to the CPMG refocusing pulse train to ensure that the initial magnetizations reflect the relative populations of the exchanging states (59). The experiments performed at 18.8 T utilized the phase cycle proposed by Yip and Zuiderweg to suppress artifacts due to off-resonance effects (60). The effective relaxation rate, *R*_2_, at a given CPMG refocusing frequency was determined from 2 data points (61). Relaxation dispersions were sampled using 18 to 24 refocusing frequencies, *ν*_cp_. The sign of the chemical shift difference between the exchanging conformations in the apo state was determined using the HSQC/HMQC approach (62). The relaxation data have been deposited in the Biological Magnetic Resonance Data Bank (BMRB) as entries 52174 (apo) (47) and 52175 (lactose, all concentrations) (50): DOI: 10.13018/BMR52174; DOI: 10.13018/BMR52175.

### Data Analysis.

NMR data were processed using NMRPipe (63). The processing protocol involved a solvent filter, cosine-squared apodization function, zero filling to twice the number of points in both dimensions, and a polynomial baseline correction in the ^1^H dimension. Peak integration and error analysis was performed using PINT (64, 65). Errors in the extracted *R*_2_ values were estimated from the S/N using error propagation.

The relaxation dispersion data of apo galectin-3C were analyzed using CPMGfit v2.42, an in-house Matlab program. The exchange parameters of the Carver-Richards two-state exchange model were fitted to the relaxation dispersion data (24, 66):

[1] $$R_{2}+R_{20}+R_{\mathrm{ex}}\left(1/\tau \right),$$

in which

[2] $$R_{ex}\left(1/\tau \right)=\frac{1}{2}\left\{k_{ex}-\frac{1}{\tau }\text{cos}h^{-1}\left(D+\cos h\left(\eta_{+}\right)-D\_\cos h\left(\eta_{-}\right)\right)\right\},$$

[3] $$D_{\pm }=\frac{1}{2}\left\{\pm 1+\;\left(\psi +2\Delta \omega^{2}\right)+{\left(\psi^{2}+\zeta^{2}\right)}^{1/2}\right\},$$

[4] $$\eta_{\pm }=\frac{\tau }{\sqrt{2}}{\left[\left\{\pm \psi +{\left(\psi^{2}+\zeta^{2}\right)}^{1/2}\right\}\right]}^{1/2},$$

and *ψ* = *k*_ex_^2^ – Δ*ω*^2^, *ζ* = –2Δ*ωk*_ex_(1–2*p*_minor_), *k*_ex_ = *k*_1_ + *k*_*−*1_ is the sum of the forward and reverse rate constants, Δ*ω* = Δ*δ* *γ* *B*_0_ is the chemical shift difference between the exchanging conformations, where *γ* is the gyromagnetic ratio of the nuclear spin and *B*_0_ is the static magnetic field strength, *R*_20_ is the average limiting value of the relaxation rate constant for processes other than chemical exchange, *p*_minor_ is the population of the minor (less populated) conformational state, which is related to the major conformational state by *p*_major_ = 1−*p*_minor_, and *τ* = 1/(2ν_cp_) is the spacing between refocusing pulses in the CPMG train.

Relaxation dispersion data for apo galectin-3C were initially modeled by residue-specific fits of the Carver–Richards equations. The statistical significance of each fit was assessed by also fitting the data to a constant *R*_20_ value (i.e., modeling a flat relaxation profile, indicating the absence of exchange), and the *F*-test was used to discriminate between models by rejecting the simpler model at the level *P* < 0.01 (67). In a subsequent step, residues with similar residue-specific exchange parameters were fitted simultaneously to yield a single, global set of parameters *k*_ex_ and *p*_minor_. Errors in the fitted parameters were estimated from 1,000 synthetic datasets created using Monte–Carlo simulations (68).

To fit the ^15^N CPMG relaxation dispersions of galectin-3C with variable amounts of lactose present, we constructed a four-state model based on the Bloch–McConnell equation (46), in which state 1 is the ligand-free, closed ground state; state 2 is the ligand-free, open high-energy state resembling the ligand-bound state; state 3 is the ligand-associated, closed state; and state 4 is the ligand-bound, open state (Fig. 1). This model includes both the CS (states 1-2-4) and IF (states 1-3-4) pathways. The exchange parameters determined from the experiments on apo galectin-3C, i.e., *k*_12_ and *k*_21_, were fixed when fitting the four-state model. δ_1_ was fixed to the value measured in the ^15^N HSQC spectrum of apo galectin-3C (given that *p*_2_ ≪ *p*_1_, the contribution from δ_2_ on the apo chemical shift is insignificant). δ_2_ and δ_3_ were fitted while δ_4_ was calculated from *p*_3_, *p*_4_, and the chemical shift measured in the ^15^N HSQC spectrum of lactose-saturated galectin-3C: δ_lac_ = *p*_3_δ_3_ + *p*_4_δ_4_. *R*_20_ was fitted individually for each residue, but globally over all lactose concentrations.

We introduced two parameters *ρ*_off_ and *ρ*_close_ describing the ratios of pairs of corresponding rates along the two pathways. *ρ*_off_ describes the ratio of the ligand off-rate constants from states 3 and 4, i.e., *ρ*_off_ = *k*_off,3_/*k*_off,4_, where *k*_off,3_ and *k*_off,4_ relate to the IF and CS pathway, respectively. *ρ*_close_ describes the ratio of the rates going from the open conformation to the closed conformation along the CS and IF pathways, i.e., *ρ*_close_ = *k*_21_/*k*_34_ (Fig. 1). Furthermore, we assumed that the on-rate constant of lactose is the same for the CS and IF pathways, which is reasonable given that the NMR relaxation experiment reports on chemical shift changes between states 1 and 3, or 2 and 4, induced by ligand binding to the binding site or its proximity.

Prior to minimization of the target function, we performed extensive grid searches on the parameters *ρ*_off_, ranging from 30 to 1,000; *ρ*_close_, 0.001 to 1,000; and *k*_on_, (8 to 300) × 10^6^ M^−1^ s^−1^, while the chemical shift differences and *R*_20_ were optimized. The combination of parameters yielding the lowest χ^2^ was taken as input for the minimization algorithm. At each iteration, the full relaxation dispersion curve was simulated for all residues, using the current values of the fitted model parameters in the Bloch–McConnell equations, and χ^2^ was evaluated. Errors in the fitted parameters were estimated from Monte–Carlo simulations using 500 synthetic datasets normally distributed by the experimental SDs (68). The Matlab code for fitting the relaxation dispersion data is available on GitHub (https://github.com/Akke-group/4 _ state_fits.git) (69).

We estimated the diffusion-limited on-rate constant for lactose binding based on the relationship (21):

[5] $$k_{\mathrm{on},D}=4\pi \;r\;D\;N_{A}\times 10^{3},$$

where *r* ≈ 20 Å is the distance between the centers of ligand and protein in the bound complex, *N*_A_ is Avogadro’s number, and *D* is the sum of the diffusion constants for lactose, *D*_lac_ = 0.57 10^−9^ m^2^s^−1^ (70), and galectin-3C, *D*_P_ = 0.126 10^−9^ m^2^s^−1^ as calculated using HydroNMR (71).

The fluxes through the CS pathway (*F*_CS_) and the IF pathway (*F*_IF_) were calculated as (20)

[6] $$F_{CS}={\left(\frac{1}{k_{12}\left[P_{1}\right]}+\frac{1}{k_{24}\left[P_{2}\right]\left[L\right]}\right)}^{-1},$$

[7] $$F_{IF}={\left(\frac{1}{k_{13}\left[P_{1}\right]\left[L\right]}+\frac{1}{k_{34}\left[P_{3}L\right]}\right)}^{-1},$$

where [*P*_1_] and [*P*_2_] are the protein concentrations of states 1 and 2, respectively, [*L*] is the free ligand concentration, [*P*_3_*L*] is the concentration of the protein–ligand complex in state 3, and *k*_AB_ is the rate constant going from state A to B. Thus, the flux through a given pathway is the inverse of the sum of the waiting times at each intermediate state.

## Supplementary Material

Appendix 01 (PDF)

## Data Availability

NMR relaxation rate constants data have been deposited in BMRB (52174 (47) and 52175 (50)). The Matlab code for fitting the relaxation dispersion data is available on GitHub (https://github.com/Akke-group/4_state_fits.git) (69).
