# Supplementary material for: Ligand-induced protein transition state stabilization switches the binding pathway from conformational selection to induced fit
Source: Proc Natl Acad Sci U S A. 2024 Mar 25;121(14):e2317747121. doi: 10.1073/pnas.2317747121 (PMC10998626; doi:10.1073/pnas.2317747121)
Supplement: Supplementary file 1 — Appendix 01 (PDF) [file pnas.2317747121.sapp.pdf]

PNAS

Supporting Information for
Ligand-induced protein transition state stabilization switches the binding pathway from conformational selection to induced fit

Olof Stenström[a], Carl Diehl[a], Kristofer Modig, and Mikael Akke*

Division of Biophysical Chemistry, Center for Molecular Protein Science, Department of Chemistry, Lund University, P.O. Box 124, SE-221 00 Lund, Sweden

[a] Present address: SARomics Biostructures AB, Medicon Village, SE-223 81 Lund, Sweden

*Corresponding author: Mikael Akke

Email: mikael.akke@bpc.lu.se

This PDF file includes:

Supporting text
Figures S1 to S3
Tables S1 to S4
SI References

Supporting Information Text

Apo Galectin-3C Transiently Adopts a Conformation Similar to the Ligand-Bound Conformation. The fact that seven residues in the binding site experience a common exchange process raises the question whether there might be residual lactose present in the apo sample that would result in exchange between a major population of the apo state and a minor population of the lactose-bound state. The purification of galectin-3C includes separation on a lactosyl-sepharose affinity chromatography column, followed by elution using 200 mM lactose. In preparing the apo-Gal3C NMR sample, affinity chromatography is followed by repeated buffer exchange to reduce the total lactose concentration to 0.1 μM, which corresponds to a population of lac-Gal3C of 0.07%, as calculated from the previously determined dissociation constant, K_d = 231 ± 15 μM (1). Conversely, the minor population observed in the CMPG experiments is 5%, corresponding to a total lactose concentration of 31 μM, which is two orders of magnitude greater than that expected. It should be noted that K_d was determined by isothermal titration calorimetry (ITC) under conditions nearly identical to those used in the present NMR experiments; specifically, the buffer composition and temperature are both identical in the ITC and NMR experiments, while the protein concentration differs by a factor of 3. Based on these considerations it appears highly unlikely that the exchange in apo-Gal3C is due to lactose binding. To verify that the observed exchange is not due to binding of residual lactose, we subjected the sample to repetitive ultrafiltration that should further reduce the concentration of lactose by a factor of 10^4. Following this treatment, relaxation dispersion experiments were repeated on the re-purified sample at 14.1 T. Compared to the experiments performed prior to ultrafiltration, fewer residues showed reliable dispersions, as a consequence of reduced S/N arising from protein losses during ultrafiltration. Nonetheless, significant relaxation dispersion remains for the majority of residues showing exchange in the original sample. Again, a set of residues in the binding site (N174, K176, E184, E185, and Q187) were grouped and fit to a common process, yielding k_{ex} = 826 ± 151 and p_M = 0.93 ± 0.02. Thus, the fitted parameters are identical within errors to those determined for the original apo-Gal3C sample. We conclude that the observed exchange in apo-Gal3C does not arise from lactose binding, but rather reports on intrinsic conformational exchange between a major ground state and a minor high-energy state.

Mapping Binding Pathways by Relaxation Dispersion Measurements at Variable Ligand Concentration. The following set of equations defines the 4-state binding model including both the IF and CS pathways. The 3 global parameters are the on-rate for ligand binding, $k_{on} = k_{13} = k_{24}$, the ratio of the rate constants for ligand release from states 3 and 4, $\rho_{off} = k_{off,3}/k_{off,4}$, and the ratio of the rate constants for protein conformational change from open to closed in each pathway, $\rho_{close} = k_{21}/k_{43}$. All rate constants (and relative populations) can be calculated from these parameters and the dissociation constant, K_d, measured by ITC, together with k_{12} and k_{21}, determined from CPMG dispersion experiments on the apo state.

$$k_{on} = k_{on1} = k_{on2} = k_{13} = k_{24} \quad \text{(S1)}$$

$$\rho_{off} = \frac{k_{off3}}{k_{off4}} \quad \text{(S2)}$$

$$\rho_{close} = \frac{k_{21}}{k_{43}} \quad \text{(S3)}$$

$$k_{off3} = k_{off4}\, \rho_{off} \quad \text{(S4)}$$

$$k_{34} = \frac{k_{43} k_{off3} k_{12} k_{on}}{k_{on} k_{off4} k_{21}} = \frac{\rho_{off} k_{12}}{\rho_{close}} \quad \text{(S5)}$$

$$k_{43} = \frac{k_{21}}{\rho_{close}} \quad \text{(S6)}$$

$$K_d = \frac{([O]+[C])[L]}{[OL]+[CL]} \quad \text{(S7)}$$

$$k_{off4} = K_d * k_{on} \left(\frac{k_{12} + \frac{k_{21}}{\rho_{off}}}{k_{12} + k_{21}} \right) \quad \text{(S8)}$$

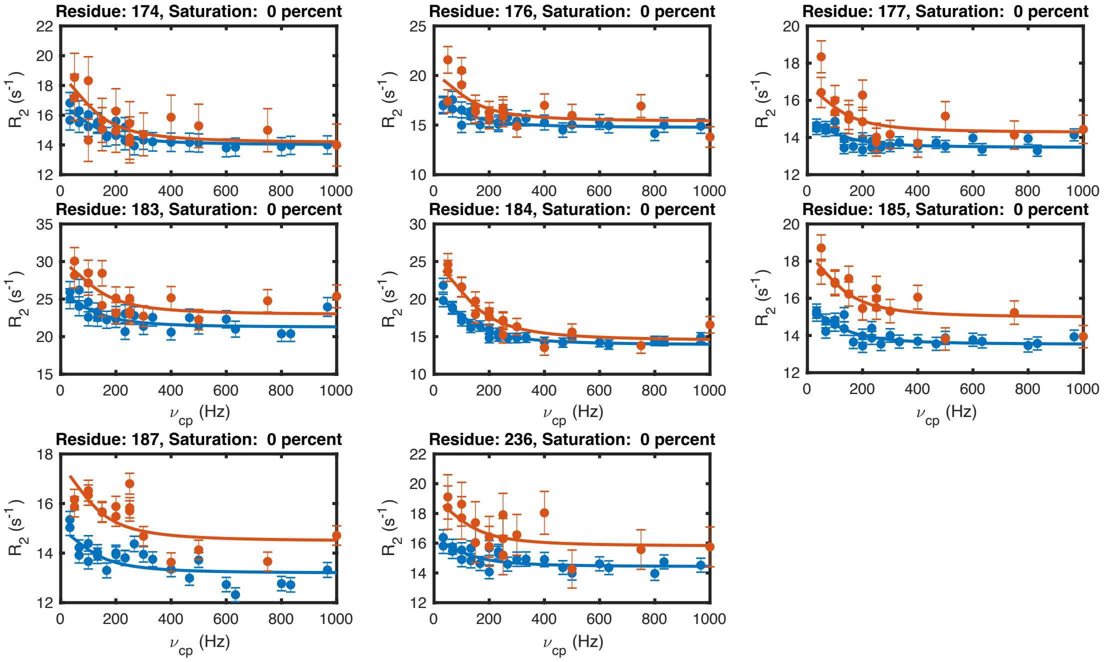

Figure S1a. ^{15}N CPMG relaxation dispersions for residues in the binding pocket for apo galectin-3C for magnetic field strengths of 18.9 (red) and 14.1 T (blue).

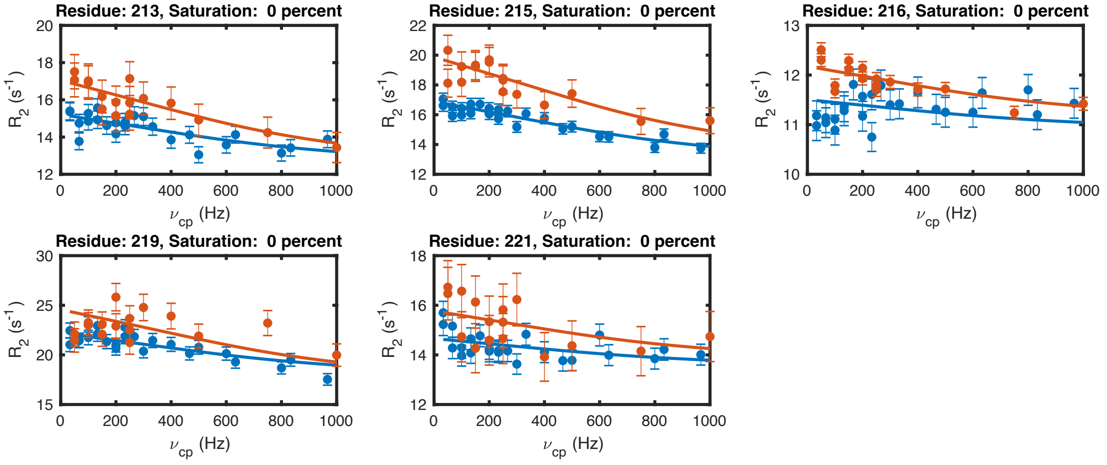

Figure S1b. ^{15}N CPMG relaxation dispersions for residues on the backside, relative to the ligand binding site, in apo galectin-3C for magnetic field strengths of 18.9 (red) and 14.1 T (blue).

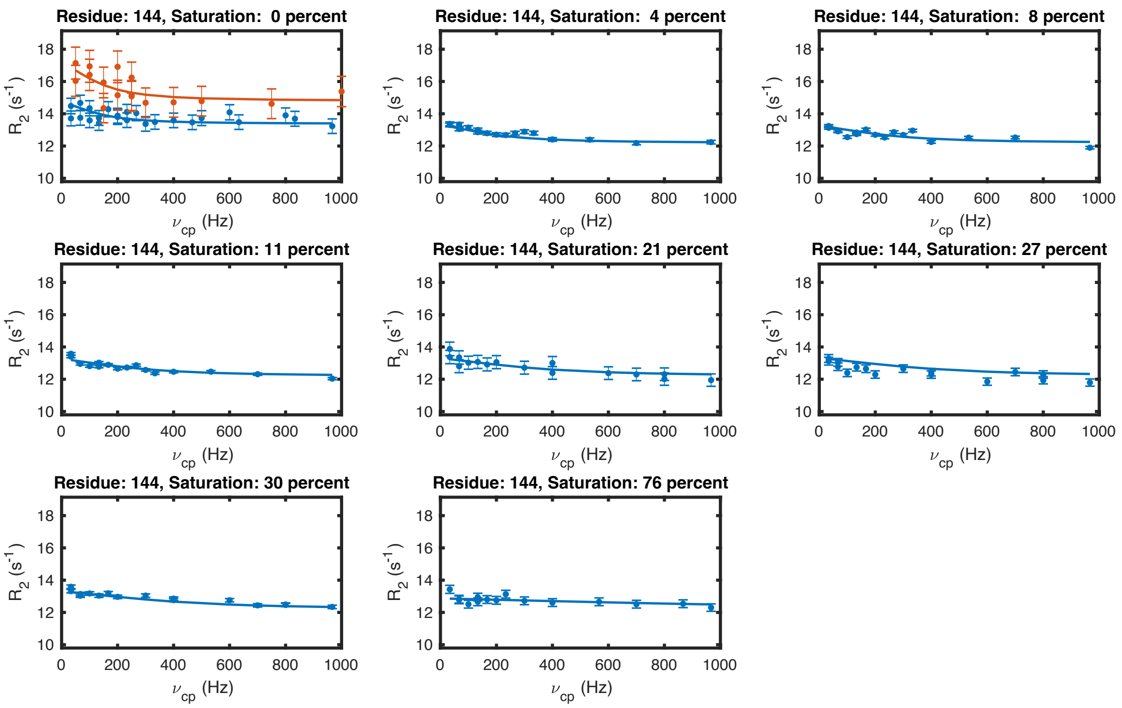

Figure S2a. ^{15}N CPMG dispersions for R144 at different lactose concentrations. Blue and red circles represent experimental data and blue and red lines are fitted relaxation dispersion curves at 14.1 and 18.8 T, respectively.

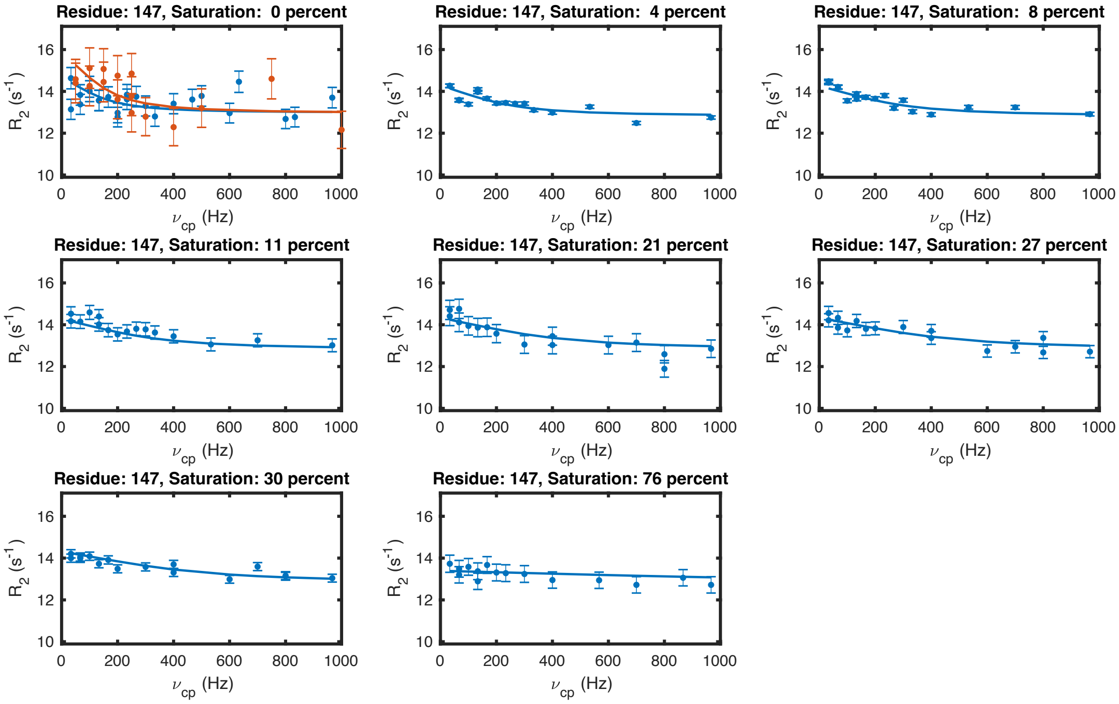

Figure S2b. [15]N CPMG dispersions for L147 at different lactose concentrations. Blue and red circles represent experimental data and blue and red lines are fitted relaxation dispersion curves at 14.1 and 18.8 T, respectively.

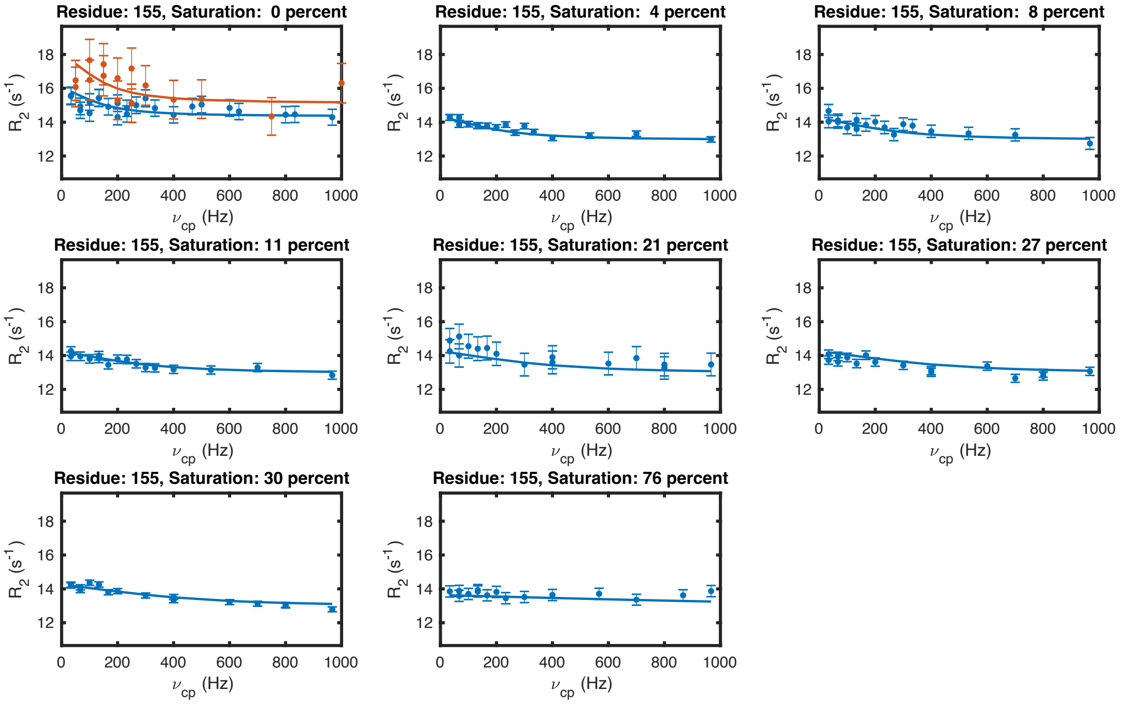

Figure S2c. ^{15}N CPMG dispersions for V155 at different lactose concentrations. Blue and red circles represent experimental data and blue and red lines are fitted relaxation dispersion curves at 14.1 and 18.8 T, respectively.

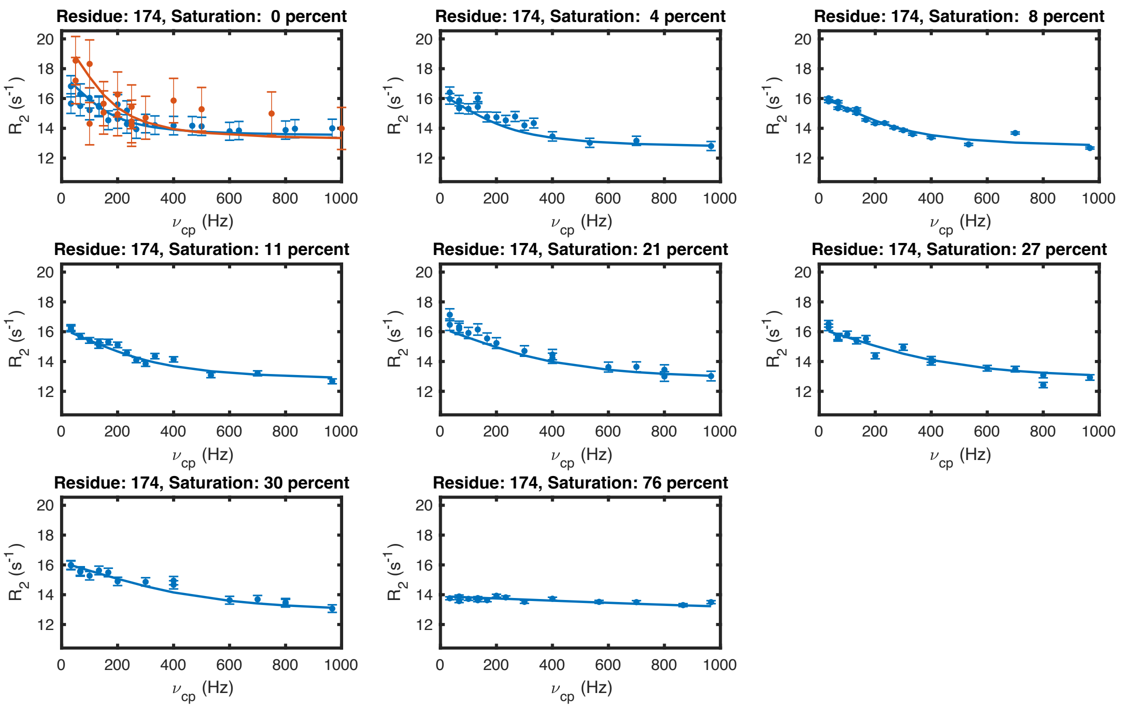

Figure S2d. ^{15}N CPMG dispersions for N174 at different lactose concentrations. Blue and red circles represent experimental data and blue and red lines are fitted relaxation dispersion curves at 14.1 and 18.8 T, respectively.

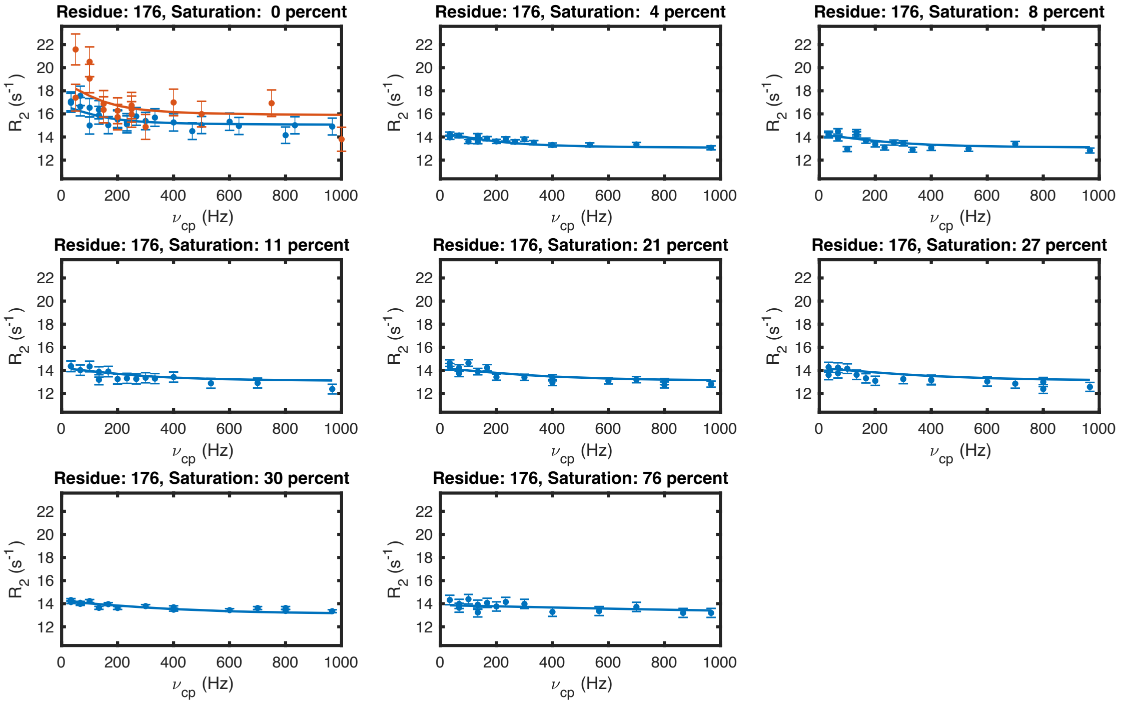

Figure S2e. ^{15}N CPMG dispersions for K176 at different lactose concentrations. Blue and red circles represent experimental data and blue and red lines are fitted relaxation dispersion curves at 14.1 and 18.8 T, respectively.

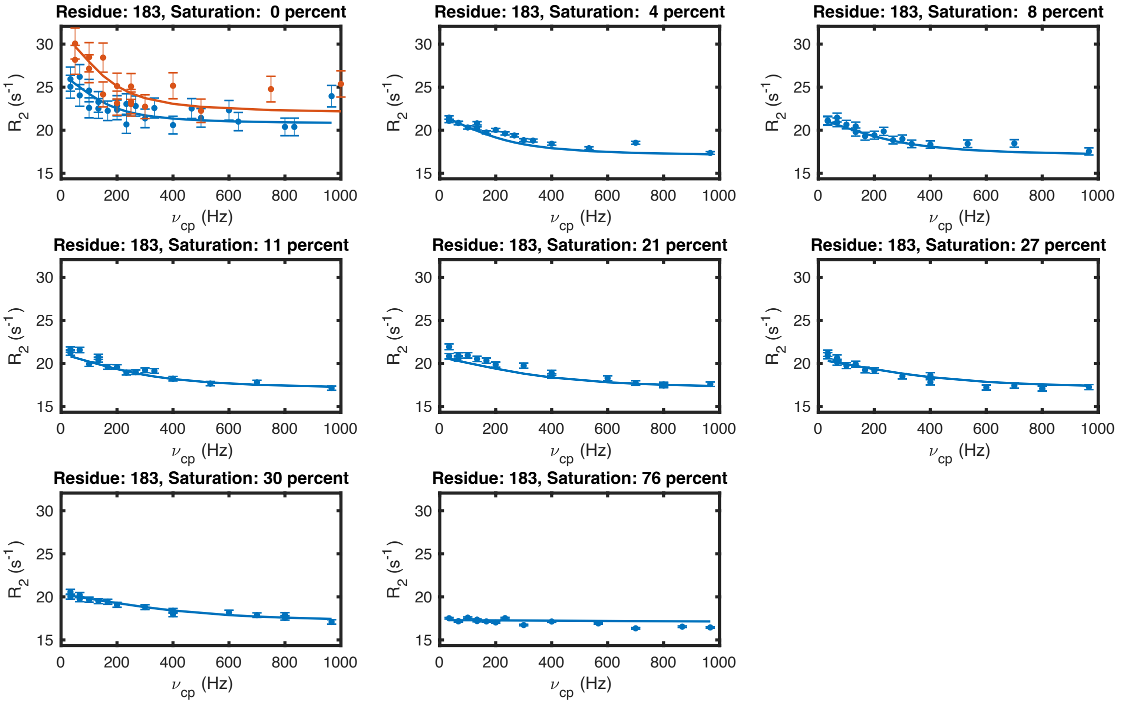

Figure S2f. ^{15}N CPMG dispersions for R183 at different lactose concentrations. Blue and red circles represent experimental data and blue and red lines are fitted relaxation dispersion curves at 14.1 and 18.8 T, respectively.

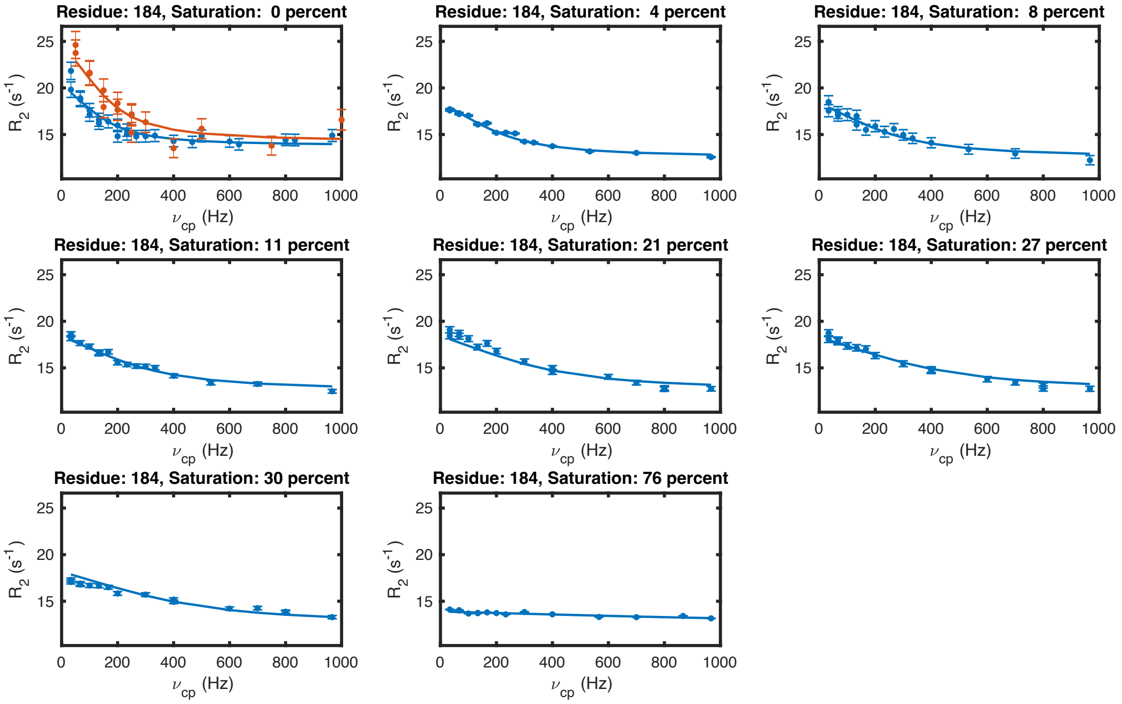

Figure S2g. ^{15}N CPMG dispersions for E184 at different lactose concentrations. Blue and red circles represent experimental data and blue and red lines are fitted relaxation dispersion curves at 14.1 and 18.8 T, respectively.

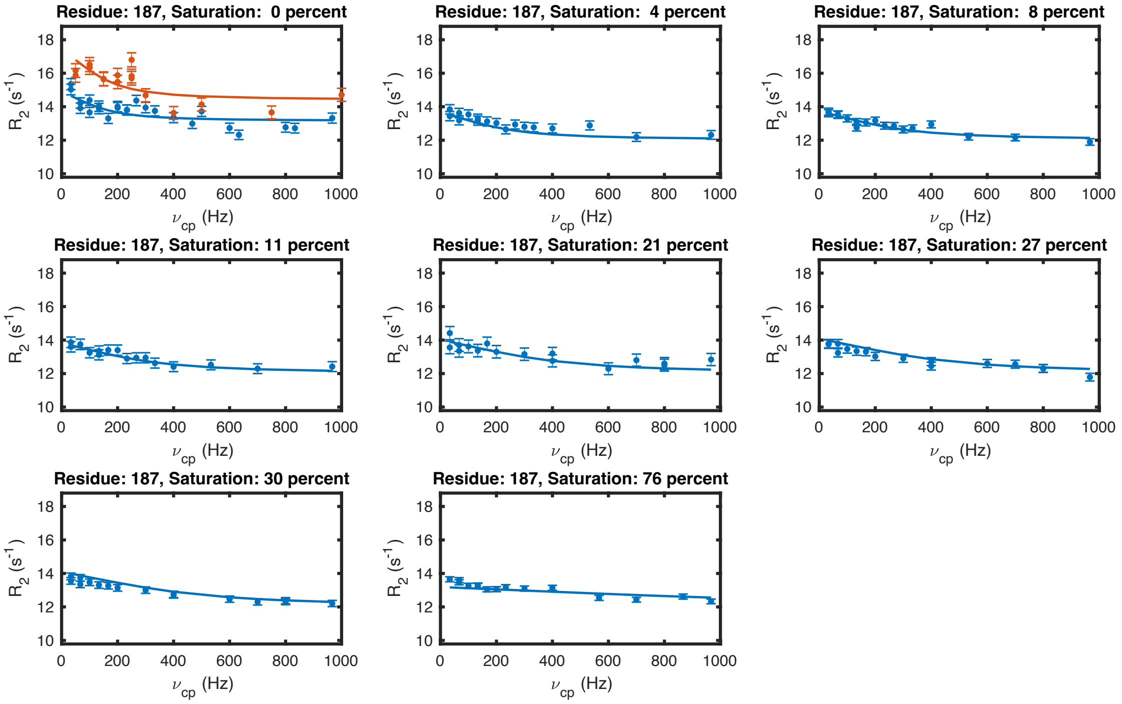

Figure S2h. ^{15}N CPMG dispersions for Q187 at different lactose concentrations. Blue and red circles represent experimental data and blue and red lines are fitted relaxation dispersion curves at 14.1 and 18.8 T, respectively.

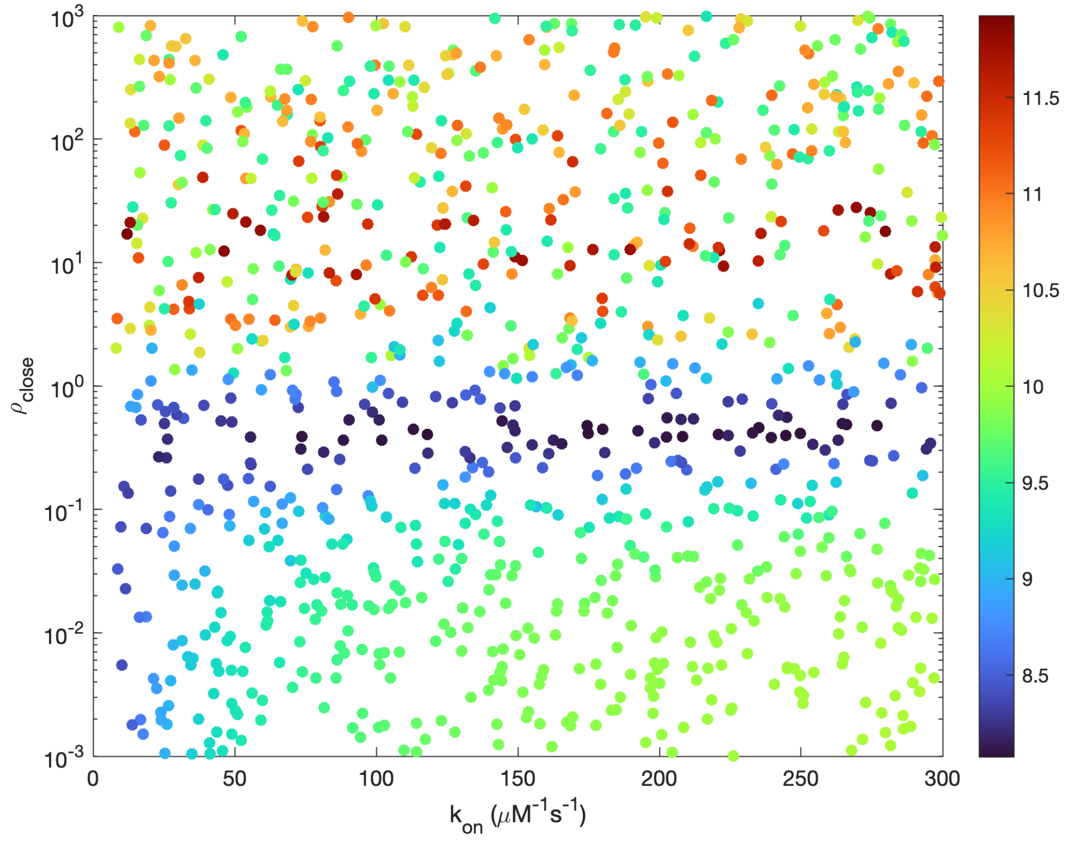

Figure S3a. χ^2 space resulting from grid search of the global parameters k_{on}, ρ_{close} and ρ_{off} projected onto the k_{on}–ρ_{close} plane. χ^2 is color coded from dark blue at low values via green and yellow to red at high values.

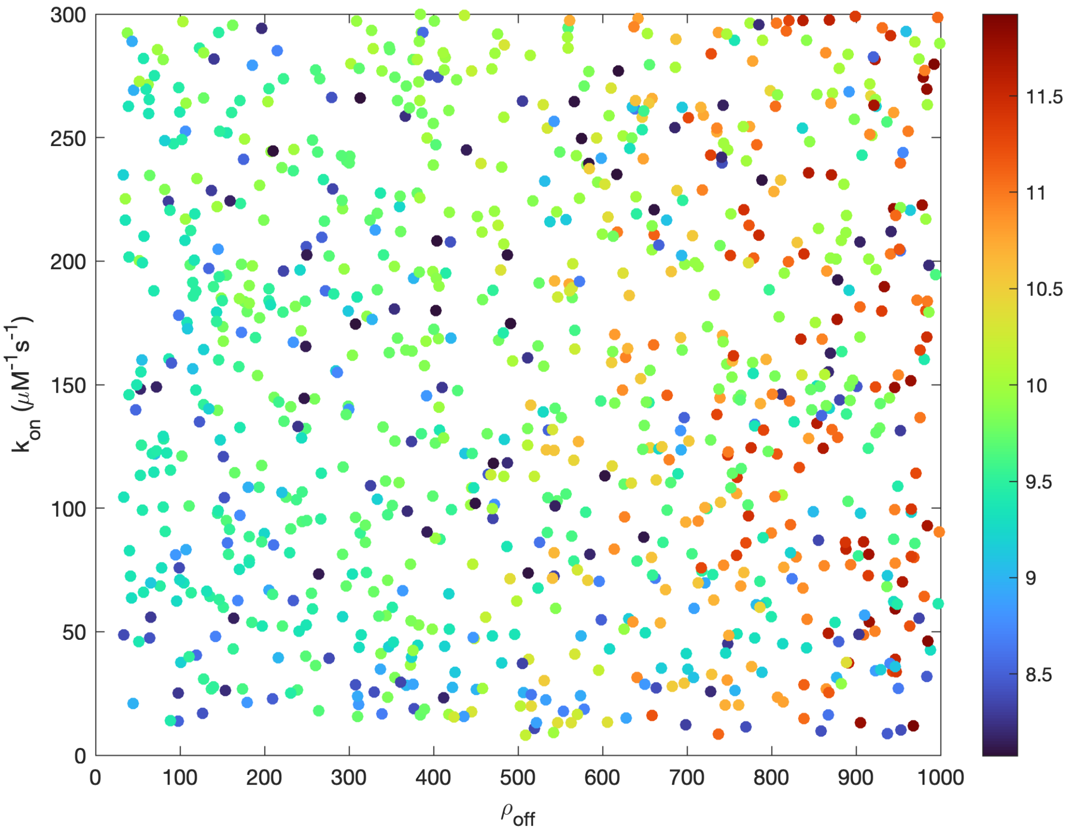

Figure S3b. χ^2 space resulting from grid search of the global parameters k_{on}, ρ_{close} and ρ_{off} projected onto the ρ_{off}–k_{on} plane. χ^2 is color coded from dark blue at low values via green and yellow to red at high values.

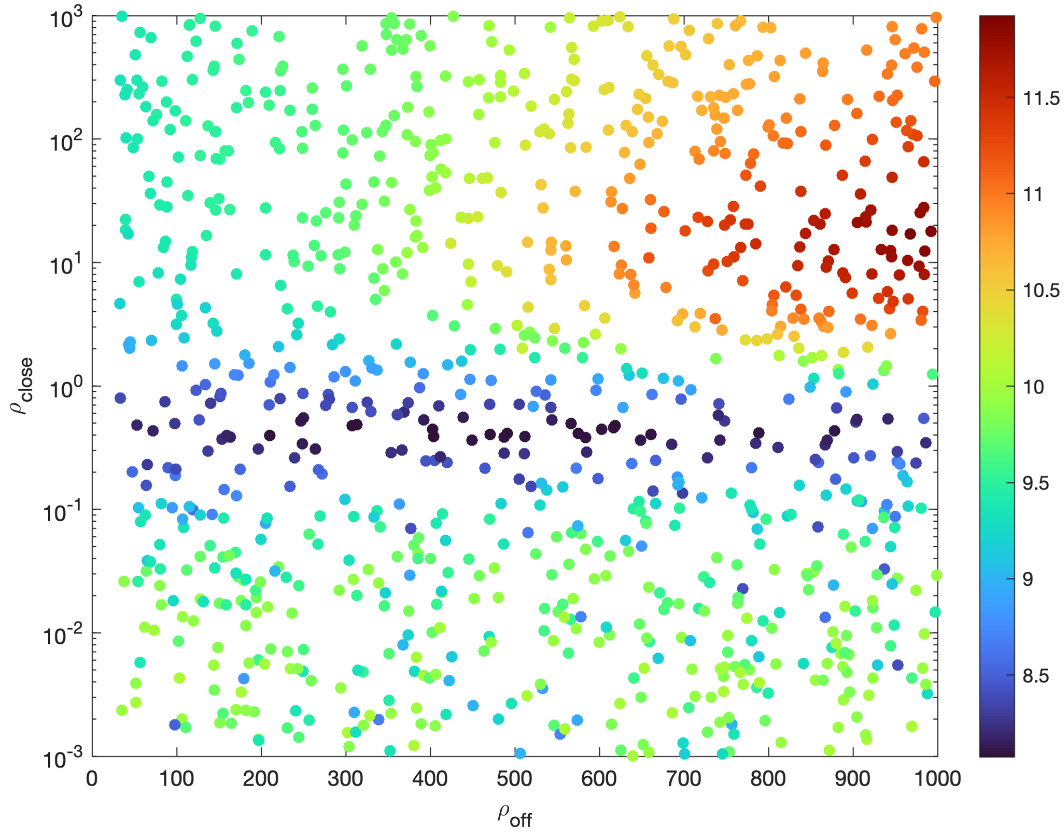

Figure S3c. χ^2 space resulting from grid search of the global parameters k_{on}, ρ_{close} and ρ_{off} projected onto the ρ_{off}–ρ_{close} plane. χ^2 is color coded from dark blue at low values via green and yellow to red at high values.

Table S1. Fitted global parameters for exchange in apo galectin-3C.

Group	Grouped residues	k_{ex} (s^{-1})	p_A
1	N174, K176, L177, R183, E184, E185, Q187, and I236	769 ± 79	0.96 ± 0.02
2	V189, V213, D215, A216, L219, and Y221	(4.8 ± 0.8) × 10^3	0.99 ± 0.01

Table S2. Fitted residue-specific parameters for exchange in apo galectin-3C.

Residue	$\Delta\delta_{CPMG}$ (ppm)
N174	0.61 ± 0.10
K176	–0.63 ± 0.11
L177	0.44 ± 0.07
R183	–0.81 ± 0.15
E184	1.02 ± 0.18
E185	0.52 ± 0.09
Q187	0.49 ± 0.08
I236	–0.50 ± 0.09

Table S3. Chemical shifts resulting from 4-state fits of ^{15}N CMPG relaxation dispersion data

Residue	δ(state 1) (ppm)	δ(state 2)	δ(state 3)	δ(state 4)
R144	117.9 ± 0.01	117.5 ± 0.01	118.6 ± 0.16	117.7 ± 0.01
L147	122.1 ± 0.01	121.7 ± 0.01	122.3 ± 0.11	121.9 ± 0.01
V155	121.5 ± 0.01	121.0 ± 0.02	122.0 ± 0.16	121.3 ± 0.01
N174	119.7 ± 0.01	119.0 ± 0.01	119.8 ± 0.10	119.3 ± 0.01
K176	130.5 ± 0.01	131.0 ± 0.02	129.5 ± 0.21	130.7 ± 0.01
R183	124.1 ± 0.01	125.0 ± 0.01	125.6 ± 0.19	124.6 ± 0.01
E184	126.1 ± 0.01	125.2 ± 0.01	125.5 ± 0.04	125.5 ± 0.01
Q187	121.9 ± 0.01	121.4 ± 0.02	122.6 ± 0.17	121.6 ± 0.01

Table S4. Summary of sample concentrations and degrees of saturation

Volume ligand added (μl)	Conc ligand solution (mM)	Total ligand conc (mM)	Total protein conc (mM)	Saturation (%)
0		0	0.44	0
1	8.75	0.029	0.206	3.9
2	8.75	0.058	0.205	7.7
3	8.75	0.086	0.205	11
6	8.75	0.166	0.204	21
8	8.75	0.217	0.203	27
11	8.75	0.248	0.302	30
14	34.8	0.946	0.321	76

SI References

1. C. Diehl, *et al.*, Protein flexibility and conformational entropy in ligand design targeting the carbohydrate recognition domain of galectin-3. *J Am Chem Soc* **132**, 14577–14589 (2010).
